# Tactile Estimation of Molded Plastic Plates Based on the Estimated Impulse Responses of Mechanoreceptive Units

**DOI:** 10.3390/s18051588

**Published:** 2018-05-16

**Authors:** Lisako Nobuyama, Yuta Kurashina, Kei Kawauchi, Koji Matsui, Kenjiro Takemura

**Affiliations:** 1 Graduate School of Science for Open and Environmental Systems, Keio University, Yokohama 223-8522, Japan; lisako@keio.jp; 2 Department of Mechanical Engineering, Keio University, Yokohama 223-8522, Japan; kurashina@mech.keio.ac.jp; 3 Kato Tech Co., Ltd., Kyoto 601-8447, Japan; k-kawauchi@keskato.co.jp (K.K.); k-matsui@keskato.co.jp (K.M.)

**Keywords:** tactile sensor, human tactile perception, mechanoreceptive units, sensory evaluation

## Abstract

This study proposes a tactile estimation method of molded plastic plates based on human tactile perception characteristics. Plastic plates are often used in consumer products. The tactile evaluation plays an important role in product development. However, physical quantities not taking into account human tactile perception have been employed in previous tactile estimation procedures. Hence, in this study, we adopted the vibrational thresholds of the mechanoreceptive units—FA I, FA II, SA I and SA II—for stimuli detection and developed a tactile estimation method for plastic plates that clarified the mechanoreceptive units related to tactile sensation. The developed tactile sensor consists of a base and a silicone rubber pad that contains strain gauges in it. We detected vibration during touch by the sensor and calculated the estimation of the firing values of the cutaneous mechanoreceptors, which are the essential data obtained by humans during tactile perception, in comparison to the amplitude spectrum of the vibration with the threshold amplitude of each mechanoreceptive unit. Simultaneously, we calculated the relationship between the normal and tangential forces recorded while the sensor ran over the samples. As a result of stepwise linear regression analysis using these values as explanatory variables, the evaluation scores for *Soft* were successfully estimated using the firing value of FA II and the relationship between normal/tangential forces, and the evaluation scores for *Rough* were estimated using the SA I firing value.

## 1. Introduction

Plastic materials are often used for the outer packaging of consumer products such as cameras, personal computers, and instrumental panels of automobiles. Appropriate design of the tactile sensation of such products may provide additional value to the product itself, in addition to its original functionality [1]. However, the tactile sensation of plastic parts has yet to be assessed compared with that of cloth or cream. Additionally, tactile estimation of plastic surfaces is a crucial matter in industrial production from the viewpoint of quality control of mass-produced consumer products manufactured in different factories.

There are a number of research studies on the tactile estimation of products with plastic and other materials [2,3,4,5,6,7,8,9,10]. Kawasegi et al., for example, investigated the relationship between the tactile sensory responses and the physical properties of the surfaces of molded plastic samples with textures at the micrometer scale [4]. Ramalho et al. developed a probe that can measure the friction coefficient between the probe and fabrics, and they obtained a correlation between the slippery/smoothness sensations and the friction coefficient [5]. Thieulin et al. developed an artificial finger to measure the friction and acoustic vibrations that occurred when the device slid on the surface of paper [6]. They showed that there was a correlation between the feature quantity from acoustic vibration that they defined, and the softness evaluated by the subjects. Song and Hu et al. developed a finger-shaped tactile sensor based on a thin PVDF film for surface texture measurement by imitating human active texture perception processes, detected the vibration from the sensor, and classified the outcomes for five different types of linen [7,8]. Chen et al. measured the vibration data obtained when an artificial finger ran over a sample. The vibration was detected by the sound wave of the conductive liquid that was used in the artificial finger [9].

The experimental data obtained in the previous research can be classified into three categories, namely, (a) the physical properties of the samples; (b) the data derived from the event caused by the interaction between sensors and samples, such as the friction coefficient; and (c) the data derived from the processing of the recorded sensor data, such as the peak value of vibration. Such data is frequently related to the tactile evaluation directly. However, in an actual human tactile perception process, the phenomena occurring in the finger are perceived by the mechanoreceptors as stimuli [11].

In this study, when stimuli are subsequently triggered and send induced impulse signals to the brain, we assumed that humans would collectively interpret that as the tactile sensation. Correspondingly, in our effort to evaluate the tactile sense of an object, it would be appropriate to emulate this process. Therefore, in this study, we develop a tactile estimation method for molded plastic samples based on the tactile perception mechanism in humans.

## 2. Materials and Methods

### 2.1. Plastic Samples

**Figure 1:**
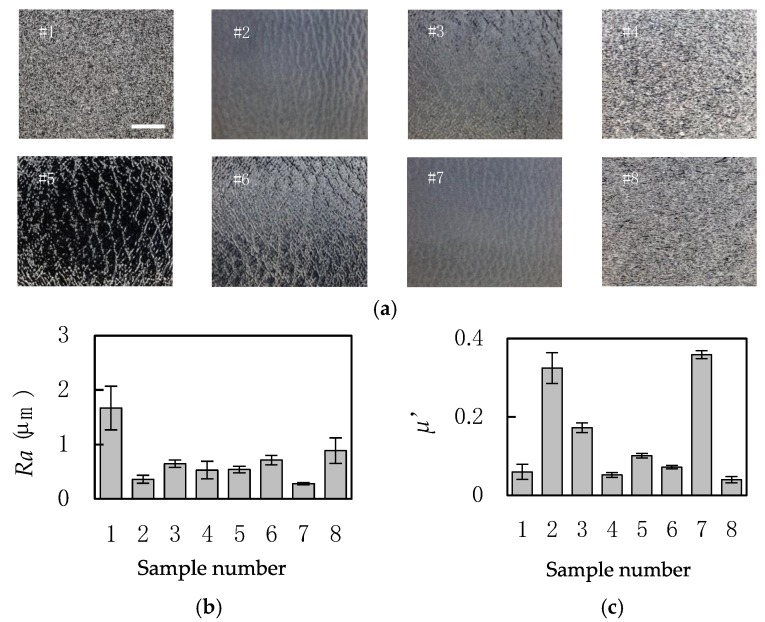
The information of plastic test samples. (**a**) The enlarged views (Scale bar: 5 mm); (**b**) arithmetic average roughness, *Ra* (mean ± SD, *n* = 10); and (**c**) dynamic friction coefficient, *µ*′ (mean ± SD, *n* = 10). Materials of the plates are: #1; polystyrene, #2; unknown, #3; polypropylene, #4; polyethylene, #5; polycarbonate, #6; polymethyl methacrylate, #7; unknown, #8; polyethylene.

Eight plastic plates, shown in Figure 1, are used in this study as test samples. The arithmetic average roughness values of the samples, *Ra*, were measured using DektakXT (Bruker Corporation, Billerica, MA, USA). The dynamic friction coefficient, *µ*′, was measured using KES-SE with a 10 mm^2^ piano-wire sensor (Kato Tech Co. Ltd., Kyoto, Japan).

### 2.2. Sensory Evauation of Plastic Samples

To obtain the tactile sense scores of different plastic samples, a sensory evaluation test was conducted with human volunteers. Note that the test protocol was approved by The Bioethics Board of the Faculty of Science and Technology, Keio University. The subjects received a thorough explanation of the test methods and then signed an informed consent form before participating in the study. The sensory evaluation was performed under 25.4 ± 0.8 °C and 61.7 ± 3.8% RH with the participation of 48 healthy adults (24 males and 24 females), aged 22.3 ± 1.4 (between 21 and 28) years old. We employed a semantic differential method with a seven-step unipolar scale. Note that evaluated words were 13 Japanese adjectives (cf. Table S1). During the test, each sample was put in a box so that visual information was excluded.

For the evaluation, words were classified using principal component analysis (PCA) [12,13] based on the evaluation scores using SPSS (Version 22, International Business Machines Corp., Armonk, NY, USA). The conditions for detecting the principal components in PCA include the criteria that: (i) the eigenvalue of each PC should be greater than unity; and (ii) the cumulative contribution rate is greater than 0.75. If these conditions were not satisfied, we deleted one of the evaluated words that had the lowest PC loading among all the evaluated words for each PC. This deletion process was repeated until the above conditions were satisfied.

### 2.3. Tactile Sensing System and Experimental Conditions

**Figure 2:**
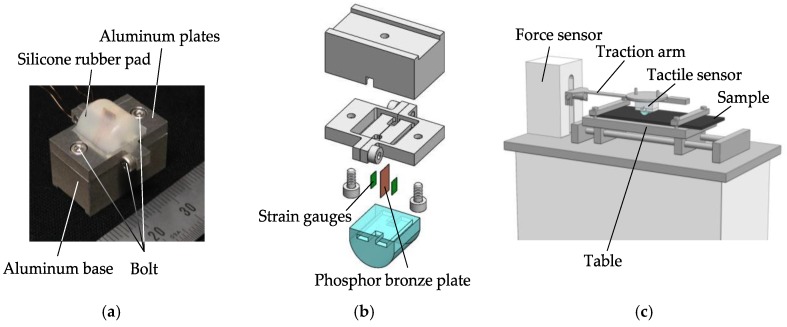
Tactile sensing system with developed tactile sensor. (**a**) Actual image of the sensor; (**b**) exploded view drawing of the sensor; and (**c**) overall view of the sensing system. The drawing in (**c**) shows the tactile sensor runs over a sample as a result of the sliding of the table. The strain gauges of the sensor detect vibration, while the force sensor measures the tangential force.

We developed a tactile sensing system capable of detecting vibration, while a tactile sensor ran over a sample. Figure 2a,b shows the actual image and an explored view drawing of the tactile sensor, respectively. Two strain gauges glued on a phosphor bronze plate are embedded in a silicone rubber pad to detect vibration induced on the sensor. The outputs from the strain gauges are acquired through a dynamic strain amplifier (DPM–913B, Kyowa Electronic Instruments Co., Ltd., Tokyo, Japan). Figure 2c shows the entire sensing system, in which the tactile sensor mentioned above is attached to the traction arm of the friction tester (KES-SE, Kato Tech Co., Ltd., Kyoto, Japan). Upon testing, the normal force, *N*, between the tactile sensor and the sample can be adjusted by placing weights on the traction arm. As the sample table of the KES-SE friction tester moves horizontally, the tactile sensor runs over the sample. The vibration data induced from the relative movement of the sensor and the sample can then be measured through the strain gauges. In addition, a force sensor connected to the traction arm detects the tangential force, *F*.

Conditions of vibration information measurement are as follows: The running speed of the tactile sensor, *V*, was 10 mm/s, the running distance was 30 mm and the normal force, *N*, applied between the tactile sensor and the sample was 0.52 N.

### 2.4. Estimation Method for Firing Values of Mechanoreceptive Unit

There are four mechanoreceptors in our glabrous skin (cf. Figure S1)—Meissner corpuscles (FA I), Pacinian corpuscles (FA II), Merkel disks (SA I), and Ruffini endings (SA II) [14,15,16]. They constitute mechanoreceptive units with corresponding neurons. The mechanoreceptive units respond to mechanical stimuli induced based on vibration inputs, and fire nerve impulses to the neuron. The relationships between the physiological threshold of the amplitudes of the stimuli for firing and the respective frequencies, are reported for each unit [15], as summarized in Figure S2 [17]. Using the experimental data plotted in Figure S2, we could approximate the threshold line, *L*, on the logarithmic chart for each mechanoreceptive unit as,

(1) $${\log L}_{\mathrm{FA}\;I}=\{\begin{array}{ll} \log499-0.890\log f, & \mathrm{if}\;f_{\mathrm{FA}\;I,1}<f\leq f_{\mathrm{FA}\;I,2}\\ \log52.1+0.0119\log f, & \mathrm{if}\;f_{\mathrm{FA}\;I,2}<f\leq f_{\mathrm{FA}\;I,3} \end{array},$$

(2) $${\log L}_{\mathrm{FA}\;\mathrm{II}}=\{\begin{array}{ll} \log1690-1.93\log f, & \mathrm{if}\;f_{\mathrm{FA}\;\mathrm{II},1}<f\leq f_{\mathrm{FA}\;\mathrm{II},2}\\ -5.00+\log2.00+1.39\log f, & \mathrm{if}\;f_{\mathrm{FA}\;\mathrm{II},2}<f\leq f_{\mathrm{FA}\;\mathrm{II},3} \end{array}$$

(3) $${\log L}_{\mathrm{SA}\;I}=\{\begin{array}{ll} \log43.5-0.545\log f, & \mathrm{if}\;f_{\mathrm{SA}\;I,1}<f\leq f_{\mathrm{SA}\;I,2}\\ \log0.787+0.683\log f, & \mathrm{if}\;f_{\mathrm{SA}\;I,2}<f\leq f_{\mathrm{SA}\;I,3} \end{array}$$

(4) $${\log L}_{\mathrm{SA}\;\mathrm{II}}=\{\begin{array}{ll} \log501-0.783\log f, & \mathrm{if}\;f_{\mathrm{SA}\;\mathrm{II},1}<f\leq f_{\mathrm{SA}\;\mathrm{II},2}\\ \log17.1-0.0340\log f, & \mathrm{if}\;f_{\mathrm{SA}\;\mathrm{II},2}<f\leq f_{\mathrm{SA}\;\mathrm{II},3} \end{array}$$

where, *L*_FA I_, *L*_FA II_, *L*_SA I_, and *L*_SA II_, are the thresholds for FA I, FA II, SA I, and SA II, respectively, and *f* is the frequency of the vibration stimulus. The parameter that expresses the frequency range for each equation can be found in Table 1. This parameter indicates the effective range of each mechanoreceptive unit and the inflection point. Each mechanoreceptive unit responds or fires if the intensity of mechanical stimulus surpasses the corresponding threshold line.

Considering the above characteristics, we estimate the firing value of each mechanoreceptive unit, i.e., running the tactile sensor, and then compare the obtained vibration data with the aforementioned threshold line in the frequency domain. Hereon, the intensity of firing of the mechanoreceptive unit (m) is represented by *I*_m_.

First, we transformed the acquired vibration data from the time domain to an amplitude spectrum, *P*, in the frequency domain using FFT, implemented in MATLAB (MATLAB 2016a, Math Works Inc., Natick, MA, USA) at a sampling frequency of 10 kHz, and with the use of the Hamming window. In addition, to compare the amplitude spectrum with the vibration detection thresholds, the threshold lines should be transformed from displacement to amplitude spectral units. To accomplish this, the conversion coefficient, *D*, was determined as follows. We selected the sample that had the lowest arithmetic average roughness, *Ra* value (henceforth referred to as *Ra*_r_), as the reference sample, assuming that we can detect its value. The frequency, *f*_r_, corresponding to *Ra*_r_ was then obtained from the running speed and the average length of the roughness curve element of a reference sample (henceforth defined as *RSm*_r_) as,

(5) $$f_{r}=V/RSm_{r}.$$

**Figure 3:**
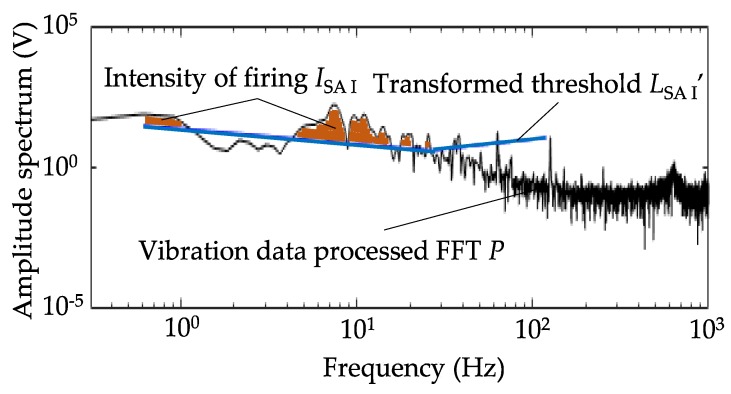
The conceptual diagram for calculation of the intensity of firing of SA I. The orange colored area between the vibration data and *L*_SA I_′ corresponds to *I*_SAI_.

Using this frequency, the conversion coefficient, *D*, could be calculated as,

(6) $$D=A/2Ra_{r},$$

where, *A* represents the amplitude spectrum value of the vibration data in the frequency domain at a frequency *f*_r_. Note that, the vibration detection thresholds of mechanoreceptive units were determined using peak-to-peak values, while the amplitude spectrum was determined using zero-to-peak amplitude values. Correspondingly, *Ra*_r_ doubled in the equation. Using *D*, the thresholds for the mechanoreceptive units (m), *L*_m_, are transformed as,

(7) $${L_{m}}^{\prime }=D\times L_{m},$$

in the amplitude spectral domain. *L*_m_′ is then superimposed on the vibration data in the frequency domain to calculate the intensity of firing, *I*_m_, as the area between the vibration data and *L*_m_′, whereby the vibration data is superior to *L*_m_′, as expressed below.

(8) $$\Delta P_{m,i}=\{\begin{array}{ll} \log P_{i}-\log{L_{m,i}}^{\prime }, & \mathrm{if}\;P_{i}\geq {L_{m,i}}^{\prime }\\ 0, & \mathrm{if}\;P_{i}<{L_{m,i}}^{\prime } \end{array}$$

(9) $$I_{m}=\sum_{f=f_{m,1}}^{f_{m,3}}\Delta P_{m,i},$$

where, *P_i_* is the amplitude spectrum of the *i*th data on the periodogram, and *L*_m,*i*_′ is the *i*th value of *L*_m_′ on the periodogram. Note that *f*_m,1_, *f*_m,2_, *f*_m,3_, are referenced from Table 1. Figure 3 shows the conceptual diagram of the stimulus values, representing an example of vibration data after the FFT of sample #3 and *L*_SA I_′. The colored area is the area where the vibration data are superior to *L*_SA I_′, and the area is defined as the intensity of firing.

In addition, to obtain the characteristics of the interaction between a sample and the tactile sensor, we calculated the relationship between the tangential force and the normal force, *M*, as,

(10) M=F/N

Note that the tangential force, *F*, used for this calculation is the average value recorded over a distance of 20 mm in a stable state (running distance ranged from 5 mm to 25 mm).

### 2.5. Tactile Estimation Method—Connecting Theacquired Data and the Principal Components

Since each principal component derived by the PCA, described in Section 2.2, could be represented by the evaluation word having the highest contribution to the PC, we performed a stepwise linear regression analysis to predict the scores of the words based on the calculated intensity of firing of the mechanoreceptive unit, *I*_m_, and the normal-tangential force relationship, *M*, using SPSS. The accuracy of prediction of the dependent variable was expressed as a contribution ratio, and the weights of the independent variables can be evaluated from the regression coefficients in the case of multiple regression analyses [3,18]. The stepwise condition was determined by *p* < 0.10.

## 3. Results and Discussion

### 3.1. Sensory Evaluation Results

Based on the sensory evaluation test, scores were obtained for all the evaluated words. After the first classification of words by PCA based on the evaluation scores using SPSS (cf. Table S2), we deleted words (*sticky*, *fine*, *dry*, *moist* and *warm*) according to the rules listed in Section 2.2. Then, we classified the words again (cf. Table S3), and deleted a word (*sticky*) according to the rules. Finally, we classified the words again by PCA. As a result, three PCs were extracted that related to softness, roughness, and coldness, as shown in Table 2. The cumulative contribution rate was 76.6% with the use of eight words. We selected only PC1 and PC2 to perform the following stepwise linear regression analyses for tactile estimation, since PC3 exhibited significantly smaller changes in the principal scores among all the samples (cf. Figure S3). The word with the highest contribution for each PC, i.e., *Soft* for PC1, and *Rough* for PC2, was regarded as the representative word for each PC. The scores for these words were then considered as the dependent variables for the stepwise linear regression analysis.

### 3.2. Estimated Index Values from Acquired Data

According to the surface roughness of sample #7 that had the lowest *Ra*, *Ra*_r_ can be defined to be 0.278 µm. The average length of the roughness curve element of sample #7, *RSm*_r_, was estimated to be 0.121 ± 0.0137 mm (mean ± SD, *n* = 10) by DektakXT. Therefore, by using the running speed, *V*, and *RSm*_r_, *f*_r_ was calculated to be 82.8 Hz, based on Equation (5). The amplitude spectral value, *A,* at *f*_r_ was 0.313 ± 0.0969 V (mean ± SD, *n* = 10) for sample #7. Using the means of *A* and *Ra*_r_, *D*, was calculated to be 0.564 V/µm based on Equation (6). Using *D*, the modified threshold for the mechanoreceptive unit (m), *L*_m_′, was calculated using Equation (7).

**Figure 4:**
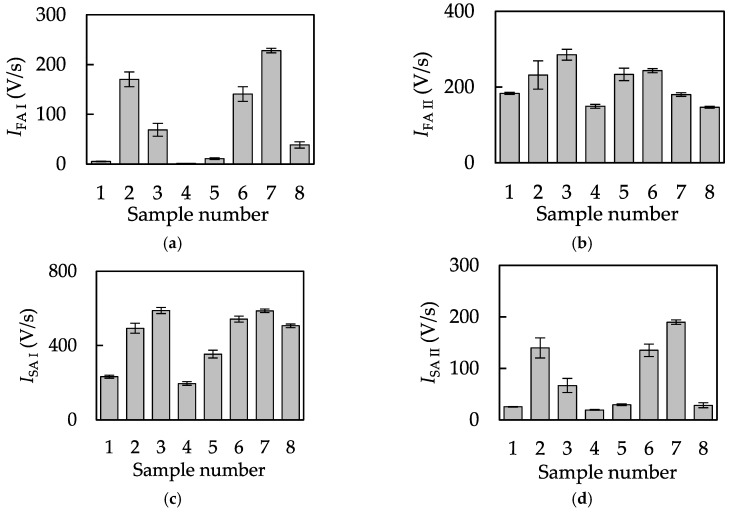
The intensity of firing of mechanoreceptive unit (m), *I*_m_. (**a**) *I*_FA I_; (**b**) *I*_FA II_; (**c**) *I*_SA I_; and (**d**) *I*_SA II_ (mean ± SD, *n* = 10).

The intensity of firing of the mechanoreceptive unit (m), *I*_m_, was then calculated using Equations (8) and (9), as shown in Figure 4. As indicated, the intensity of firing of each mechanoreceptive unit differed for different samples. Some previous studies were conducted to estimate tactile sensation using vibration information, but they only used physically featured qualities, such as the frequency of resonance vibration [19], and the peak value of vibration [9]. Neither of these markers was related to the firing nature of the mechanoreceptive units. Tactile estimation using the psychophysical thresholds of the mechanoreceptive units was also reported [20], but the firing of each mechanoreceptive unit was not estimated. In addition, the threshold lines were empirically defined. On the other hand, this study derived the intensity of each mechanoreceptive unit with appropriately defined threshold lines, and the derived intensities of the mechanoreceptive units represented the differences of test objects.

**Figure 5:**
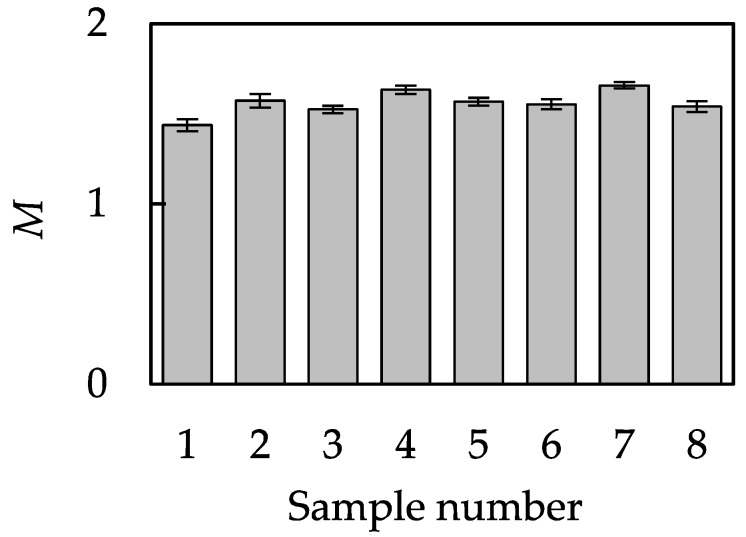
The relationship, *M,* between the tangential force *F* and load *N* (mean ± SD, *n* = 10).

Figure 5 shows the normal-tangential force relationship, *M*, of each sample. The reason *M* exceeded unity is probably due to the stick-slip phenomenon occurring between the silicone rubber pad and the samples [21]. This is the reason we do not refer to *M* as the friction coefficient.

### 3.3. Stepwise Linear Regression Analyses

**Figure 6:**
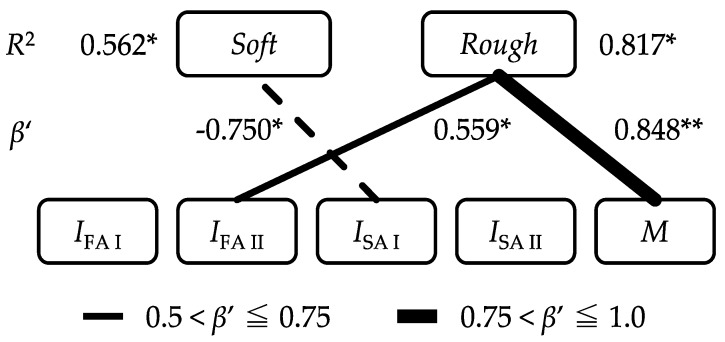
Structure of tactile perception (* denotes significance—*p* < 0.05; ** denotes significance—*p* < 0.01). The positive values of *β*′ are shown using solid lines, and the negative values using dotted lines.

Using the intensity of firing of mechanoreceptive unit (m), *I*_m_, and *M* as index values, we performed stepwise linear regression analyses to estimate the results of sensory evaluations. The results of the stepwise linear regression analyses between the dependent variables (the scores of *Soft* and *Rough*), and the explanatory variables (*I*_m_ and *M*), are shown in Figure 6. The prediction models are formulated as,

(11) $$Soft=5.59-0.002\times I_{\mathrm{SA}\;I},$$

(12) $$Rough=-16.075+0.011\times I_{\mathrm{FA}\;\mathrm{II}}+11.633\times M$$

In the regression model for *Soft*, *I*_FA I_, *I*_FA II_, *I*_SA II_, and *M* are excluded during the stepwise analysis because they fail to meet the quantifying criterion (*p* < 0.10). The usability of *I*_SA I_ was found to significantly contribute to the prediction of the determinants of *Soft* (*R*^2^ = 0.562, *p* < 0.05). Although *R*^2^ value is not high enough to conclude correlation between *I*_SA I_ and *Soft*, there is a clear linear relation between them as shown in Figure S4. *R*^2^ value only reflects the dispersion of the plots in this case. The detail of the model is shown in Table S4. SA I has the capacity to reflect spatio-temporal information [22], and is indicative of the skin response subject to its fluent deformation. The amount of skin deformation and the contact area with the sample are related to the recognition of softness. In this study, the samples were not deformed significantly based on pressure exerted by the finger, but the finger deformation was mainly caused by horizontal interactions. The interaction along the horizontal direction was derived from the stickiness of the samples, and was considered to influence the evaluation of *Soft*. However, humans detect the softness of an object by touching while changing the pressing force of the finger. Therefore, the reason that *R*^2^ of the model for *Soft* was small lays in the measurement method. The tactile sensor was pulled horizontally with a constant load and the normal force was not changed while the sensor is running over the sample. 

Additionally, in the regression model for *Rough*, *I*_FA I_, *I*_SA I_, and *I*_SA II_, are excluded based on the quantifying criterion (*p* < 0.10). Usability of *I*_FA II_ and *M* were found to significantly contribute to the prediction of the determinants of *Rough* (*R*^2^ = 0.817, *p* < 0.05). The detail of the model is shown in Table S5. In this model, it was shown that *Rough* became smaller as *I*_FA II_ became larger. It is known that there are macroscopic markers and fine roughness markers in the psychophysical aspects of tactile sense [23]. Moreover, there is a published report indicating that the tactile sensation is smoothed when low-frequency vibrations are combined with high-frequency vibrations [24]. While FA II plays a role in acceleration detection [25], *I*_FA II_ was selected for the *Rough* detection model because it concurrently responded to the vibration stimulus at a relatively higher frequency. Furthermore, the regression model for *Rough* also suggested the importance of *M* in this estimation. It is known that the normal-tangential force relationship (generally perceived as the friction coefficient) affects the surface roughness feeling of tactile sensation [26].

**Figure 7:**
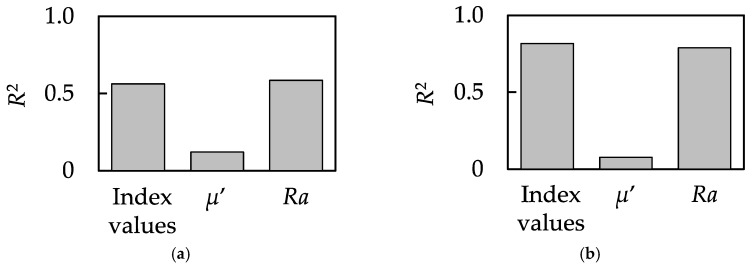
Comparison of the results of regression analyses for (**a**) *Soft* scores and (**b**) *Rough* scores using the index values, *µ*′ and *Ra*.

To confirm the effectiveness of the proposed method, we also performed single regression analyses using *Ra* and the dynamic friction coefficient, *µ*′, both of which have been extensively used for the estimation of tactile sensation [27,28]. The usability of *Ra* is found to significantly contribute to the prediction of the determinants of *Soft* (*R*^2^ = 0.586, *p* < 0.05), while the usability of *µ*′ does not. In addition, the usability of *Ra* was also found to significantly contribute to the prediction of the determinants of *Rough* (*R*^2^ = 0.788, *p* < 0.01), while the usability of *µ*′ does not. The details of the models are shown in Tables S6 and S7. Figure 7 shows the comparison of the results from linear regression analyses using the index values and the results of simple regression analyses using *Ra* and *µ*′. From this result, it was found that the *R*^2^ of the model for *Soft* and *Rough* using the index values was superior to that elicited using *µ*′ and was close in value to that elicited using *Ra*. However, the meaning of the model using *Ra* and the model using the index values are entirely different. Humans do not detect surface roughness determined for machining, but the stimuli detected by the mechanoreceptors allow the sense of tactile feeling. For this reason, the model using the index values is capable of estimating a tactile sensation based on the mechanism of human tactile perception. Furthermore, with the model using the index values proposed in this study, we clarified which receptors are strongly related to which tactile sensation. Therefore, using our method, estimating tactile sensation and clarifying the receptors involved is possible at the same time.

## 4. Conclusions

Estimation of tactile sensation is required for the development of products to improve their commercial values. For this, we developed a tactile sensing system that is capable of detecting vibration and tangential forces, while a sensor ran the tested samples. From the vibration data obtained, estimations of the firing values of the mechanoreceptive units were calculated based on the human tactile perception mechanism. Simultaneously, an estimation of the force relationship value between normal/tangential forces was calculated. We also conducted sensory evaluations to obtain the sample scores for different words under evaluation, and extracted three principal components for the tactile sensation of tested samples using principal component analyses. As a result of tactile estimations using stepwise linear regression analyses, we constructed regression equations to estimate the scores of the representative words of the two principal components from the estimated values. In conclusion, *Soft* and *Rough* scores were successfully estimated from the acquired data.

## Figures and Tables

**Table 1:** The effective frequency range for each mechanoreceptive unit.

m	*f*_m,1_ (Hz)	*f*_m,2_ (Hz)	*f*_m,3_ (Hz)
FA I	0.500	12.2	67.0
FA II	20.0	241	800
SA I	0.500	26.2	120
SA II	0.500	91.2	400

**Table 2:** Results obtained from the principal component analysis.

Evaluate Index	PC1 (Softness)	PC2 (Roughness)	PC3 (Coldness)
*Soft*	0.893	−0.0650	0.0141
*Hard*	−0.873	0.0528	0.0976
*Elastic*	0.853	−0.0617	0.0413
*Rough*	−0.0600	0.874	−0.0498
*Coarse*	−0.0711	0.824	−0.0597
*Smooth*	0.0419	−0.791	0.106
*Cold*	−0.0140	−0.0621	0.927
*Cool*	−0.0199	−0.127	0.918
Eigenvalue	2.55	2.11	1.47
Contribution rate (%)	28.7	26.2	21.6
Cumulative contribution rate (%)	28.7	54.9	76.6

## Supplementary Materials

The following are available online at http://www.mdpi.com/1424-8220/18/5/1588/s1, Figure S1: The mechanoreceptors in the human finger, Figure S2: The physiological threshold-frequency characteristics for mechanoreceptive units, (a) FA I, (b) FA II, (c) SA I, and (d) SA II. The approximate lines represent *L*_m_, Figure S3: The relationship between the principal scores of (a) PC1 and PC2, (b) PC1 and PC3, and (c) PC2 and PC3, Figure S4: The relation between the SA I index value, *I*_SA I_, and the evaluation score for *Soft*, Table S1: Words used for the sensory evaluation test (terms in brackets are in Japanese), Table S2: Results obtained from the first principal component analysis. *Sticky, fine, dry, moist* and *warm* do not meet the criteria listed in Sec. 2.2, Table S3: Results obtained from the second principal component analysis. *Sticky* does not meet the criteria listed in Sec. 2.2, Table S4: Results of the stepwise linear regression analyses between the index values and *Soft* scores, Table S5: Results of the stepwise linear regression analyses between the index values and *Rough* scores, Table S6: Comparison results of the stepwise linear regression analyses of *Soft* scores using the index values, *μ′* and *Ra*, Table S7: Comparison results of the stepwise linear regression analyses of *Rough* scores using the index values, *μ′* and *Ra*.
