# Supplementary material for: Tactile Estimation of Molded Plastic Plates Based on the Estimated Impulse Responses of Mechanoreceptive Units
Source: Sensors (Basel). 2018 May 16;18(5):1588. doi: 10.3390/s18051588 (PMC5981637; doi:10.3390/s18051588)
Supplement: Supplementary file 1 [file sensors-18-01588-s001.pdf]

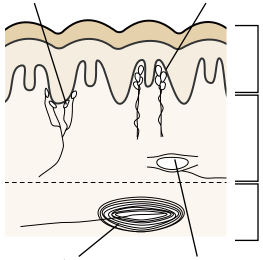

Figure S1. The mechanoreceptors in the human finger.

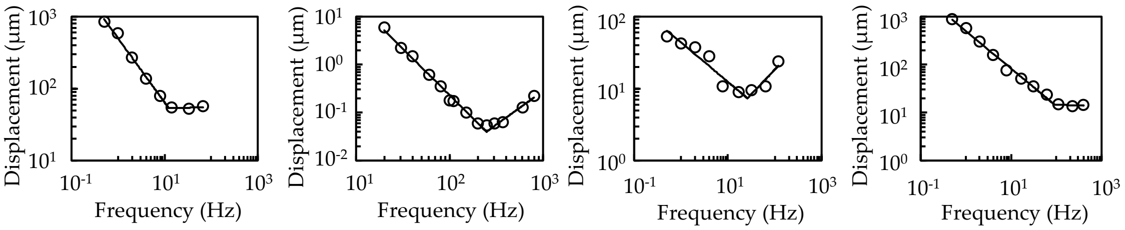

(a) (b) (c) (d)

Figure S2. The physiological threshold-frequency characteristics for mechanoreceptive units, (a) FA I, (b) FA II, (c) SA I, and (d) SA II. The approximate lines represent L_{m}.

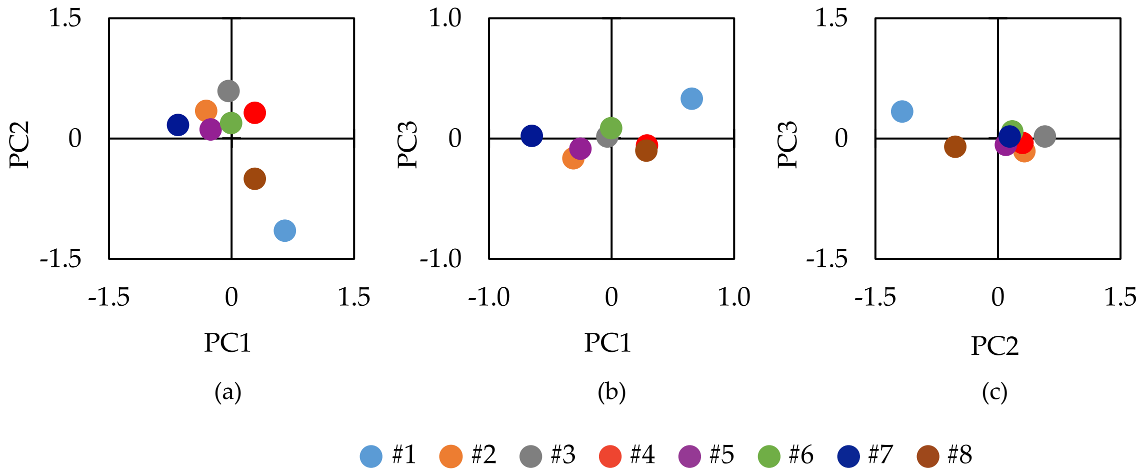

Figure S3. The relationship between the principal scores of (a) PC1 and PC2, (b) PC1 and PC3, and (c) PC2 and PC3.

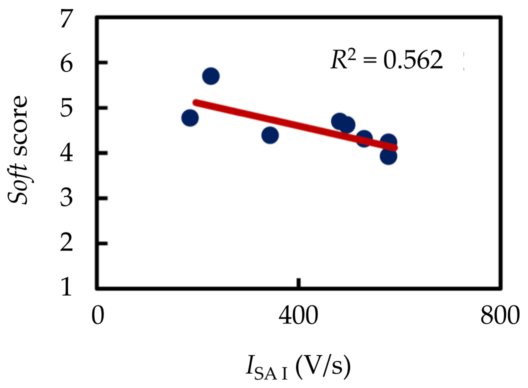

Figure S4. The relation between the SA I index value, $I_{SA\ I}$, and the evaluation score for *Soft*.

Table S1. Words used for the sensory evaluation test (terms in brackets are in Japanese).

		Evaluation words (Japanese)		
Rough (Zarazara-suru)	*Coarse (Arai)*	*Moist (Shittori-suru)*	*Sticky (Hikkakari-no-aru)*	*Soft (Yawarakai)*
Sleek (Subesube-suru)	*Fine (Komakai)*	*Dry (Sarasara-suru)*	*Hard (Katai)*	*Elastic (Danryoku-no-aru)*
Cold (Tsumetai)	*Cool (Hinyari-suru)*	*Warm (Atatakai)*		

Table S2. Results obtained from the first principal component analysis. *Sticky*, *fine*, *dry*, *moist* and *warm* do not meet the criteria listed in Sec. 2.2.

Evaluate index	PC1	PC2	PC3
Sleek	0.795	0.083	0.087
Rough	-0.766	-0.167	-0.007
Coarse	-0.765	-0.158	-0.032
Sticky	-0.638	-0.054	-0.044
Fine	0.619	-0.004	0.007
Dry	0.596	-0.192	0.147
Soft	0.005	0.885	-0.013
Hard	0.010	-0.834	0.113
Elastic	-0.003	0.831	0.021
Moist	0.293	0.551	-0.059
Cold	0.108	0.032	0.881
Cool	0.163	0.039	0.873
Warm	0.033	0.172	-0.590
Eigenvalue	3.37	2.55	1.70
Contribution rate (%)	23.7	20.0	14.9
Cumulative contribution rate (%)	23.7	43.7	58.6

Table S3. Results obtained from the second principal component analysis. *Sticky* does not meet the criteria listed in Sec. 2.2.

Evaluate index	PC1	PC2	PC3
Rough	0.831	-0.069	-0.065
Sleek	-0.787	0.045	0.110
Coarse	0.786	-0.080	-0.074
Sticky	0.726	-0.009	-0.003
Soft	-0.070	0.892	0.012
Hard	0.057	-0.872	0.099
Elastic	-0.048	0.855	0.045
Cold	-0.060	-0.014	0.927
Cool	-0.124	-0.020	0.918
Eigenvalue	2.79	2.19	1.55
Contribution rate (%)	27.6	25.6	19.3
Cumulative contribution rate (%)	27.6	53.1	72.4

Table S4. Results of the stepwise linear regression analyses between the index values and *Soft* scores.

	Unstandardized coefficient β	Standardized coefficient β'	p value	Variance Information Factor (VIF)	Model summary		
					R^2	Adjusted R^2	p value
Constant	5.59		0.00001		0.562	0.489	0.032
$I_{\mathrm{SA\ I}}$	-0.00248	-0.750	0.032	1.00			

Table S5. Results of the stepwise linear regression analyses between the index values and *Rough* scores.

	Unstandardized coefficient β	Standardized coefficient β'	p value	Variance Information Factor (VIF)	Model summary		
					R^2	Adjusted R^2	p value
Constant	-16.1		0.015		0.817	0.744	0.014
M	11.6	0.848	0.008	1.05			
$I_{\mathrm{FA\ II}}$	0.0105	0.559	0.036	1.05			

Table S6. Comparison results of the stepwise linear regression analyses of *Soft* scores using the index values, μ' and *Ra*.

	R^2	Adjusted R^2	R^2 change	p value
Index values	0.562	0.489	0.370	0.032
μ'	0.120	-0.0270	0.525	0.401
Ra	0.586	0.517	0.360	0.027

Table S7. Comparison results of the stepwise linear regression analyses of *Rough* scores using the index values, μ' and *Ra*.

	R^2	Adjusted R^2	R^2 change	p value
Index values	0.817	0.744	0.470	0.014
μ'	0.0765	-0.0775	0.964	0.507
Ra	0.788	0.752	0.462	0.003
